# Effect of Glutathione Bio-Molecule on Tooth Discoloration Associated with Silver Diammine Fluoride

**DOI:** 10.3390/ijms19051322

**Published:** 2018-04-29

**Authors:** Mahmoud Sayed, Naoko Matsui, Noriko Hiraishi, Toru Nikaido, Michael F. Burrow, Junji Tagami

**Affiliations:** 1 Cariology and Operative Dentistry, Graduate School of Medical and Dental Sciences, Tokyo Medical and Dental University, 113-8510 Tokyo, Japan; Drmahmoudmacklad@gmail.com; 2 Cariology and Operative Dentistry, Oral Restitution Department, Graduate School of Medical and Dental Sciences, Tokyo Medical and Dental University, 1-5-45, Yushima, Bunkyo-ku, 113-8549 Tokyo, Japan; nikaido.ope@tmd.ac.jp (T.N.); tagami.ope@tmd.ac.jp (J.T.); 3 Research Fellow of Japan Society for the Promotion of Science, Tokyo Medical and Dental University, 113-8510 Tokyo, Japan; hiraope@tmd.ac.jp; 4 Faculty of Dentistry, University of Hong Kong, Hong Kong, China; mfburrow@unimelb.edu.au

**Keywords:** silver diammine fluoride, glutathione, discoloration, spectrophotometer

## Abstract

This study evaluated the effect of Glutathione (GSH) bio-molecule on the reduction of enamel and dentin discoloration after application of 38% silver diammine fluoride solution (SDF). One hundred and twenty bovine teeth specimens were used. The enamel and dentin specimens were divided into three groups: (1) SDF only (control); (2) SDF followed by application of a potassium iodide solution (KI); and (3) SDF mixed with 20% GSH. Half the specimens were exposed to light and the remainder kept in dark conditions (*n* = 10) Color changes were measured using a spectrophotometer at the following time intervals: before solution application (baseline) and immediately after application, then 3, 6, 24, 48, 72 h, and 7, 10 and 14 days. SEM/EDS analysis was performed on treated enamel and dentin. Statistical analysis was done using a repeated measures ANOVA test. The spectrophotometer results showed that the SDF group exhibited the greatest color changes under both light exposed and dark conditions, while SDF + GSH group was effective in decreasing the color changes in both light and dark conditions. The SDF + KI group showed an insignificant color changes over time. SEM/EDS analysis showed different patterns for the silver crystal formation in each group (SDF, SDF + GSH, and SDF + KI group). It was concluded GSH can effectively minimize color changes after application of SDF, especially on enamel and to a lesser extent on dentin.

## 1. Introduction

Dental caries is a prevalent bacterial, multifactorial, dynamic disease caused by an imbalance between demineralization and remineralization processes of tooth structure. It is considered one of the most common diseases affecting humans [1]. Different cost effective preventive measures are needed as a mean to reduce the effects of dental caries such as pain, and loss of natural teeth [2].

Silver compounds, like silver nitrate and silver fluoride, have been used in dental treatments over many years. Their use commenced with the management of dental caries in deciduous teeth, which then progressed as a preventive agent on permanent teeth [3]. Being introduced by Yamaga et al., in the late 1960’s [4], silver diammine fluoride (SDF) is one of these silver compounds on which experiments have been conducted with promising results documented in the literature showing its ability to protect against and arrest dental caries [2,5,6].

SDF is considered as an efficient, cost effective, and non-invasive material that can be used on deciduous and permanent teeth. It has been demonstrated to have a unique anti-bacterial effect and can inhibit demineralization and increase the surface micro-hardness of the tooth structure [5]. SDF is proven to be more effective in caries prevention compared to fluoride varnish [6], where it has been shown to lead to 70% of caries lesions arresting compared to 46% for fluoride varnish. Annual and biannual applications of SDF can significantly reduce the development of new lesions, offering high effectiveness in protection against caries at low cost. [7].

However, the main drawback of SDF is the formation of dark stains on tooth structure. This unpleasant outcome limits its clinical use [8] due to aesthetic concerns.

Application of potassium iodide (KI) after SDF results in a creamy white precipitate of silver iodide (AgI), which is considered as one of the methods that can overcome the staining problem [9]. However, recently it was noticed that the color improvement with KI is only temporary and the darkening of tooth surfaces still occurs [10,11]. In addition, the use of SDF and KI together reduces free silver ions [9], which may decrease the merits of SDF dramatically, most likely over the long term.

An alternative method has been developed which may decrease staining from SDF and preserve the silver within the solution and on the substrate surface by mixing glutathione with SDF, which will be investigated in this paper

Glutathione (GSH) is considered one of the most common intracellular non-protein thiols (NPSH) that acts as a reducing agent in mammalian cells. NPSH is an anti-oxidant that can act as a metal chelator and radical quencher [12].

GSH has been previously used as a biomimetic coating on silver particles to enhance interactions with complex bio-systems and increase its water solubility [13,14].

The aim of this study was to evaluate the effect of glutathione bio-molecule on the degree of enamel and dentin discoloration after application of silver diammine fluoride (SDF), with and without light exposure at different time intervals. The null hypothesis in this study is GSH has no effect on the color changes of enamel and dentin after application of SDF.

## 2. Results

### 2.1. Spectrophotometric Measurement of Color Changes

A repeated measure ANOVA was conducted to compare the different tested groups for color changes (ΔE) of the dentin surface over a 14-day time interval. The data were obtained immediately, 3, 6, 24 and 72 h followed by 7, 10 and 14 days. Mauchly’s test indicated that the assumption of sphericity had been violated; therefore, the Greenhouse-Geisser corrected tests were used. There was a significant interaction between time and groups at F (19.517, 172.8) = 31.159, *p* ≤ 0.001. Post hoc comparisons indicated that there was a difference among the tested groups at all time periods (*p* ≤ 0.001).

**Figure 1:**
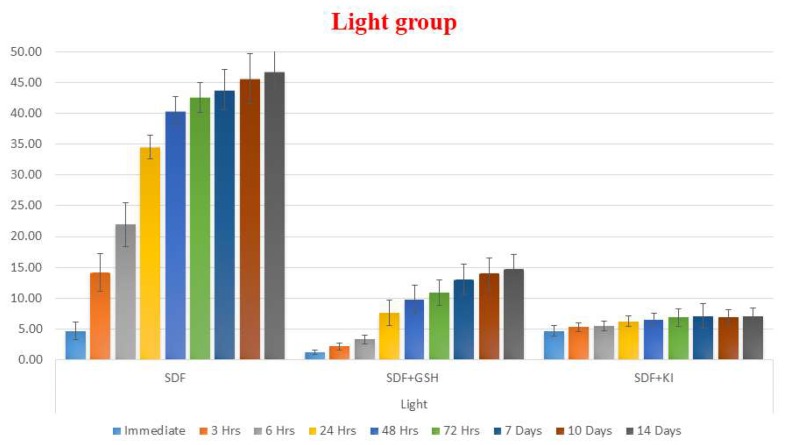
Bar chart showing mean ΔE for different tested groups for enamel substrate within each time interval under light exposed conditions.

In the light exposed enamel groups, GSH showed a strong influence in reducing the color changes (ΔE) of enamel after application of SDF, while the SDF group showed the greatest color changes compared to the other groups at all time periods. The SDF + KI group showed a minor immediate color changes which darkened insignificantly over the two weeks of observations (Table 1 and Figure 1).

**Figure 2:**
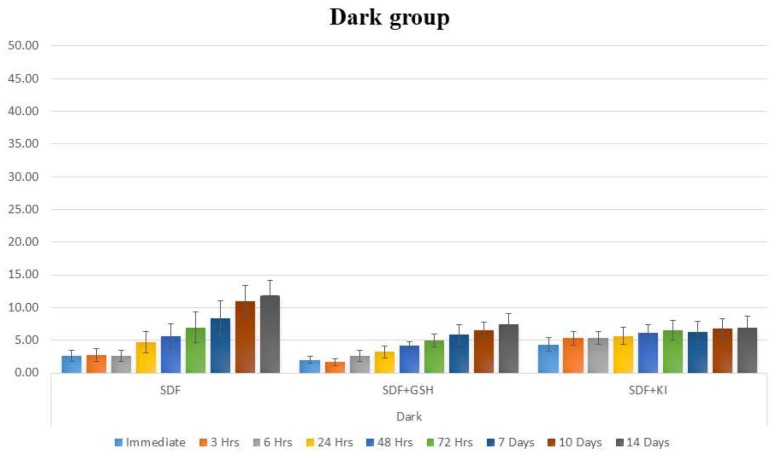
Bar chart showing mean ΔE for different tested groups for enamel substrate within each time interval under dark conditions.

The dark stored enamel groups showed the same pattern as the light exposed groups, however at lower values of ΔE with the SDF + GSH group showed improvement in the esthetic outcome (Table 2 and Figure 2). 

**Figure 3:**
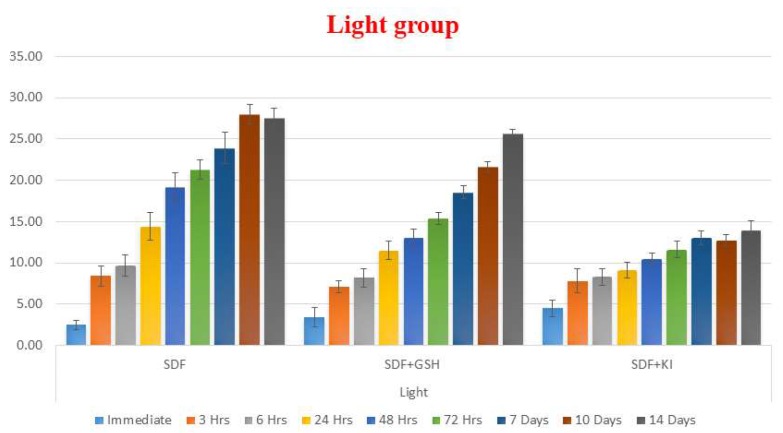
Bar chart showing mean ΔE for different tested groups for dentin substrate within each time interval under light exposed conditions.

In the dentin groups exposed to light, GSH reduced the color changes but it was less than that for enamel, while SDF also showed the highest ΔE values and SDF + KI group showed the largest change in color immediately after solution application but after two weeks, it showed the least overall change in color (Table 3 and Figure 3).

**Figure 4:**
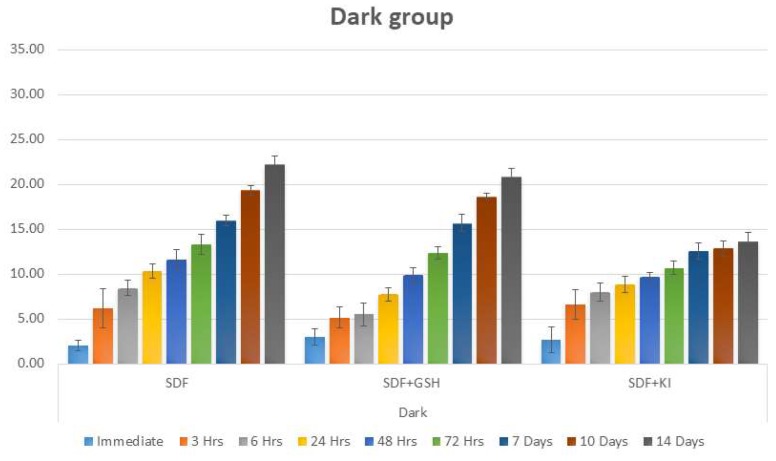
Bar chart showing mean ΔE for different tested groups for dentin substrate within each time interval under dark conditions.

Regarding the dark stored dentin group, the same pattern was noticed as the group exposed to light, but with a lower scale of ΔE values (Table 4 and Figure 4).

### 2.2. SEM Observation Results

**Figure 5:**
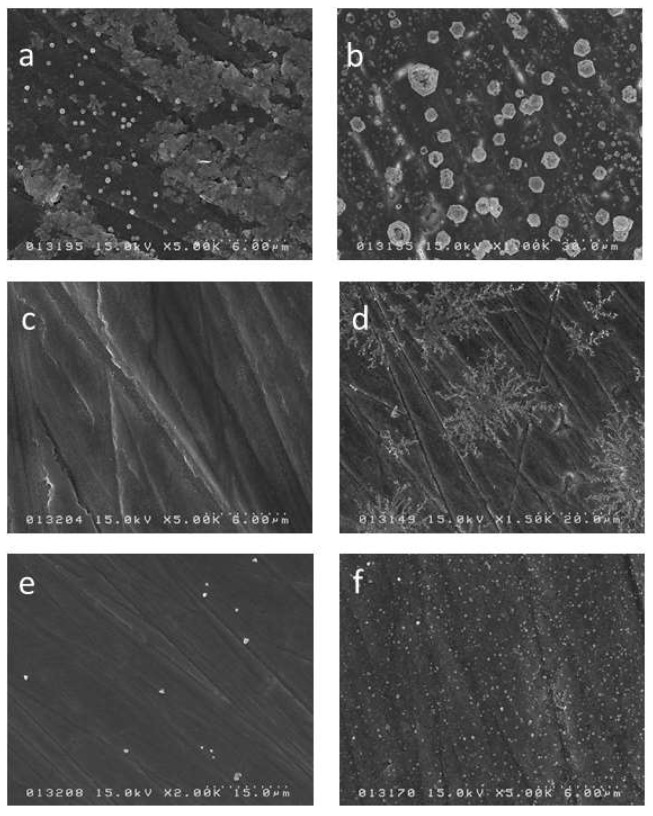
Scanning electron microscope (SEM) image for the surface of the enamel specimens: (**a**) SDF 24 h; (**b**) SDF 2 weeks; (**c**) SDF + GSH 24 h; (**d**) SDF + GSH 2 weeks; (**e**) SDF + KI 24 h; and (**f**) SDF + KI 2 weeks.

For the SDF enamel group, the SEM image showed small (less than a micrometer) separate crystals distributed over the surface while after 2 weeks the image showed greater aggregation of crystals of a larger size which were spread over the whole surface (Figure 5a,b). The SDF + GSH enamel group did not show any crystal formation after 24 h, however by 2 weeks crystal formation had occurred and displayed a radial branching pattern (Figure 5c,d). The SDF + KI enamel group showed very few crystals after 24 h, however like the other groups by 2 weeks there was a noticeable increase in crystal formation over the surface (Figure 5e,f).

**Figure 6:**
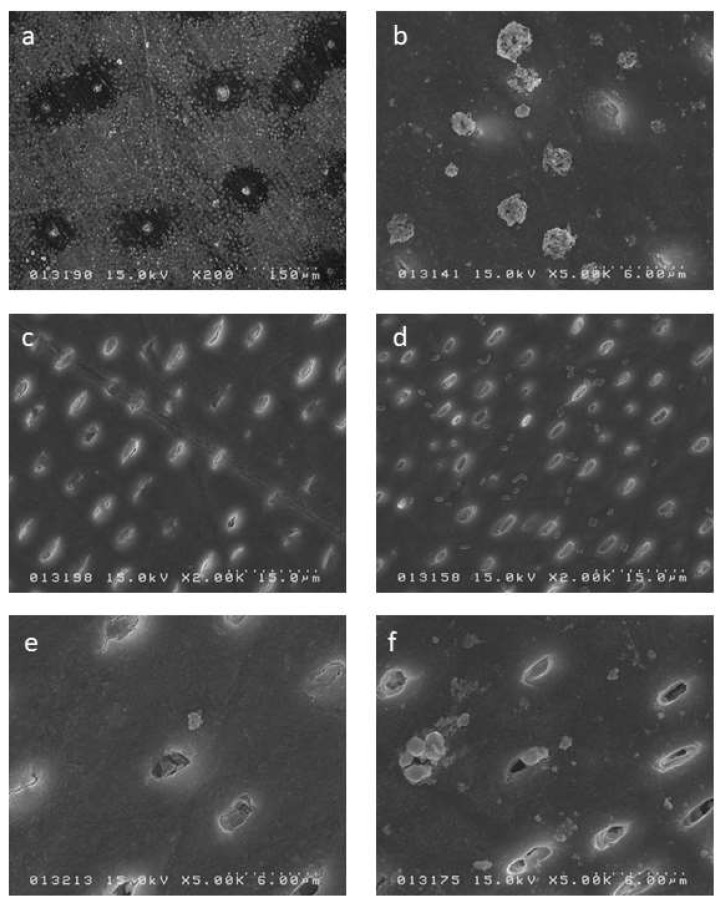
SEM image for the surface of the dentin specimens: (**a**) SDF 24 h; (**b**) SDF 2 weeks; (**c**) SDF + GSH 24 h; (**d**) SDF + GSH 2 weeks; (**e**) SDF + KI 24 h; and (**f**) SDF + KI 2 weeks.

Regarding the SDF dentin group, the SEM image showed sparse crystal formation on the surface and associated dentinal tubule occlusion, while at 2 weeks the dentin surface had larger crystal formation but this remained sparse (Figure 6a,b). The SDF + GSH dentin group showed partial occlusion of dentinal tubules with no crystal formation on surface, but at 2 weeks, crystal formation was observed over the whole surface (Figure 6c,d). The SDF + KI dentin group showed very few crystals over the surface and only partial occlusion of the dentinal tubules, while at 2 weeks the crystal formation had increased in frequency and size (Figure 6e,f).

**Figure 7:**
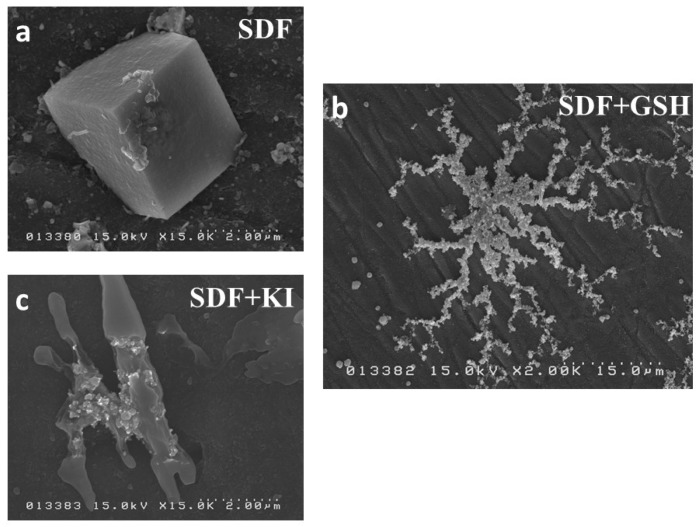
SEM image for the pattern of crystal formation: (**a**) SDF group; (**b**) SDF + GSH group; and (**c**) SDF + KI group.

Regarding the pattern of crystal formation at higher magnification, the SDF group showed typical cubic-shaped crystals (Figure 7a), the SDF + GSH group showed branching or radial-shaped aggregations of crystals (Figure 7b), while the SDF + KI group showed irregular-shaped crystal formation (Figure 7c).

### 2.3. EDS Elemental Analysis

**Figure 8:**
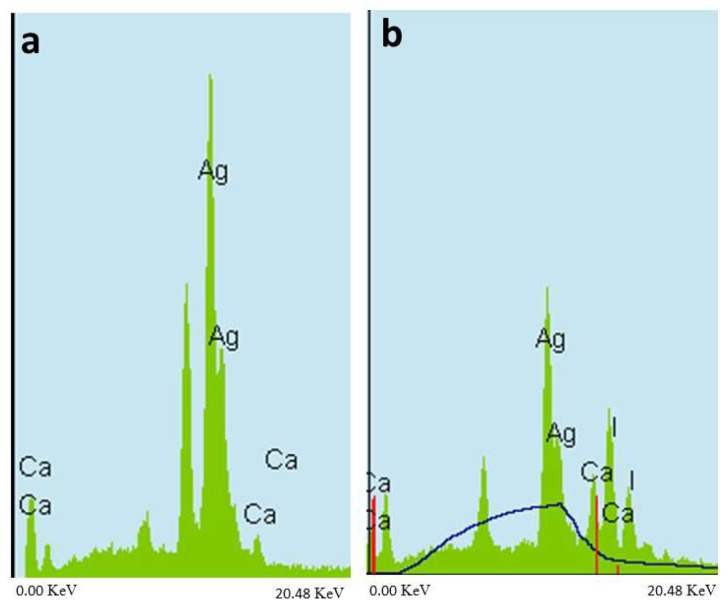
Energy dispersive X-ray spectroscopy (EDS) analysis: (**a**) SDF and SDF + GSH group and (**b**) SDF + KI group.

The EDS point analysis for the crystals formed in both the SDF group and the SDF + GSH group showed a high peak of silver, confirming that the crystals were silver (Figure 8a), while the SDF + KI group showed the highest peak for silver followed by iodine, confirming presence of silver iodide (Figure 8b). 

## 3. Discussion

SDF is a colorless solution that contains a high concentration of fluoride (44,880 ppm) and silver (25.5%). Upon application of SDF, it reacts with tooth structure to release a large quantity of free silver ions (Ag^+^). When these ions are reduced, they aggregate and precipitate resulting in the formation of dark stains on the tooth surface, which has caused esthetic concerns among practitioners and patients alike [15]. Since SDF is sensitive to light which leads to surface color changes on tooth structure, a spectrophotometer was chosen to measure the color changes as it is able to record and measure the full visible spectrum for analysis [16]. A spectrophotometer is a sophisticated digital instrument, which can measure the complex color of tooth structure numerically with high reliability and reproducibility [16].

Potassium iodide was introduced in an attempt to solve the color change problem associated with SDF. Upon application of a saturated solution of potassium iodide immediately after SDF application, silver iodide crystals are formed and precipitate on the surface as a creamy white precipitate [9]. The reaction between KI and SDF is depicted as:

Ag(NH_3_)_2_F + KI → AgI + KF + 2NH_3_

Glutathione (GSH) is a tri-peptide biomolecule and considered the best candidate with silver as it contains a thiol group (-SH) which has a high affinity for adsorption onto metal surfaces [17]. GSH forms a coat around silver particles, decreasing the aggregation of silver particles as well as controlling the rate of silver ion release (Homeostasis) [14], which may play a role in decreasing the rate of color changes of an SDF-coated tooth surface over time.

As bovine teeth are reported to resemble human teeth in many features including radio-density [18], dentin surface roughness and even more homogeneity in respect to mineral composition compared with human teeth [19]. It was considered beneficial to use bovine teeth for the evaluation of color in order to eliminate as many confounding variabilities as possible among the enamel and dentin specimens. Caries-free dentin and enamel specimens were used in this study, as the purpose was to test staining of SDF as a preventive agent. 

In case of enamel, silver (Ag^+^) and fluoride (F^−^) ions released from SDF can penetrate the surface up to 25 µm [20], and react with enamel to form mainly calcium fluoride (CaF_2_), silver phosphate (Ag_3_PO_4_) and a small quantity of metallic silver [21]. While in the case of dentin, the silver and fluoride ions are able to penetrate as much as 50–200 µm into the dentin, and react with the it to form mainly metallic silver attached to protein (silver protein) [21] as well as CaF_2_, and Ag_3_PO_4_ in a lesser amount [8].

Silver phosphate (Ag_3_PO_4_) crystals are yellow in color once formed and gradually turn dark, especially if exposed to light as the silver ions are released and reduced to metallic silver [21].

As most of the free silver ions have already reacted with the iodine salt (KI) that may be the reason for the insignificant change in color between different time intervals in this study when the tooth substrates were treated with the SDF + KI solution. However, other studies have reported significant color changes after long term storage [10,22,23].

The GSH group demonstrated a significant decrease in color changes in enamel, which showed only a slight change in color changes, whereas the change in color for dentin was slightly greater color changes. This may be because the formation of metallic silver was much less on enamel than dentin and the percentage of “20%” GSH was sufficient for enamel to minimize color changes, but for dentin it seems necessary to use an increased concentration of GSH to more effectively reduce color changes.

According to the results of the current study, GSH was more effective in reducing color changes of enamel and dentin, therefore the null hypothesis was rejected.

In this study, we mixed SDF with 20% GSH by weight. We were not able to increase the percentage of GSH to more than 20% as it was difficult to dissolve the GSH in SDF. May be with this percentage of (20%) GSH, it was difficult to completely coat all of the silver particles.

The ability of GSH to decrease the color changes of enamel and dentin may encourage the use of SDF in a greater variety of clinical situations with better esthetic outcomes than SDF alone.

Further studies are needed to better understand simpler ways on how to increase the GSH concentration in currently used SDF solutions.

## 4. Materials and Methods

This study protocol was approved by the ethics committee of Tokyo Medical and Dental University (11 December 2017) with identification code “D2013-022-02” (Institutional Research Board approval number: 725)

### 4.1. Specimens Preparation

One hundred and twenty bovine teeth specimens, free of cracks or caries (sixty enamel and sixty dentin) were used. Enamel specimens (6 × 6 mm) were prepared from the labial surface of bovine incisor crowns, while dentin blocks (6 × 6 mm) were prepared from the labial and lingual surfaces of the cervical portion of bovine incisor roots. The specimens were cut using a low-speed diamond saw (IsoMet 1000, Buehler Ltd., Lake Bluff, IL, USA) under water coolant. Specimens were embedded in acrylic resin (Unifast III, GC, Tokyo, Japan) and the surfaces were successively ground flat using 600–2000-grit silicon carbide papers (SiC) (Fuji Star, Sankyo Rikagaku, Saitama, Japan) under running water. The specimens were then ultra-sonicated in distilled water (DW) (Milli-Q water; Millipore, Billerica, MA, USA).

For each of the enamel and dentin groups, the specimens were randomly divided into three groups according to solution application. The first group received SDF treatment as a control group, the second group received SDF followed by application of a potassium iodide solution (2.36 mol/L), while the third group received SDF mixed with 20% GSH (Table 5).

### 4.2. Material Application

Materials were applied according to manufacturer instructions.

1- Thirty-eight percent SDF: applied to the tooth surface and agitated using a micro-brush for 1 min, left for 2 min and rinsed with a copious amount of distilled water for 30 s;
2- SDF followed by KI: SDF was applied to the tooth surface, immediately followed by application of a saturated KI solution until creamy white precipitates turned clear, then washed with copious amounts of distilled water for 30 s; and,
3- SDF mixed with 20% GSH: under vigorous stirring, SDF was mixed with 20% GSH by weight until the solution was clear without any precipitates. Then applied in the same manner as the SDF.

Each group was divided into two subgroups according to light exposure namely, light and dark subgroups (*n* = 10). The light groups were kept in clear containers and exposed continuously to a fluorescent light source (FBL18EXL, Mitsubishi, 18W, Tokyo, Japan) with an intensity of approximately 4 mWatts/cm^2^ and wavelength 410–460 nm, while the dark groups were kept in lightproof containers. All specimens were incubated at 37 °C.

### 4.3. Color Assessment

Color assessments of the specimens (*n* = 10) were recorded at ten time-interval points: baseline (before solution application), immediately after solution application, 3, 6, 24, 48, 72 h, and 7, 10, 14 days after application. Color was recorded using a spectrophotometer (JP7200F, Juki, Tokyo, Japan) with a wave length range of 400–700 nm. The instrument was calibrated before each examination time according to the manufacturer’s instructions. Each color was explained using the 3-dimensional CIELAB color space system (frequently denoted as L*a*b*), where L* represents brightness ranging from dark (0) to bright (100), a* describes red (+a*) to green (−a*), and the b* represents yellow (+b*) to blue (−b*). A single operator repeated color measurements three times for each specimen at each time interval and the mean values recorded. 

The color difference (ΔE) of each specimen between baseline and each time-interval point was calculated using the following equation [24]; 

ΔE = [(ΔL)^2^ + (Δa)^2^ + (Δb)^2^]^1/2^

### 4.4. .Morphology Observation

For morphological analysis of the crystals formed on specimen surfaces after solution application, a further eight samples were prepared for each group and treated in the same manner described for specimen preparation for the color measurement. Half of the specimens for each treatment group were observed after 24 h and the remaining half were observed after 2 weeks. Specimens were sputter-coated with osmium (4 nm layer thickness). The surfaces of the prepared specimens were examined under a scanning electron microscope (SEM, H-4500, Hitachi High-Technologies Corporation, Tokyo, Japan) under operating conditions of 15 kV.

### 4.5. Elemental Analysis

For elemental analysis of the treated surfaces, five additional specimens were prepared for each treatment group and prepared in the same manner described for specimen preparation for the color measurement and analyzed after 2 weeks. The specimens were sputter-coated with osmium (4 nm layer thickness). The elemental point analysis for the surfaces of the specimens was performed to detect phosphorous (P), calcium (Ca), silver (Ag) and iodine (I) ion levels via energy dispersive X-ray spectroscopy (EDS) under a scanning electron microscope (H-4500, Hitachi High-Technologies Corporation, Tokyo, Japan) under operating conditions of 15 kV.

### 4.6. Statistical Analysis

Data were analyzed for normality using the Kolmogorov-Smirnov and Shapiro-Wilk tests. Repeated Measures ANOVA test was used to compare the effect of the different materials and time intervals on mean ΔE. The test was followed by pairwise comparison with Bonferroni correction. The significance level was set at *p* ≤ 0.05. Mauchly’s and greenhouse tests were applied. Statistical analysis was performed using IBM^®^ SPSS^®^ (SPSS Inc., IBM Corporation, Armonk, NY, USA) Statistics Version 23 for Windows.

## 5. Conclusions

Glutathione bio-molecule has an effect in decreasing tooth surface color changes after application of SDF, especially on enamel and to a lesser extent on dentin.

## Figures and Tables

**Table 1:** Mean and standard deviation (SD) for the color change (ΔE) for different tested groups at all time intervals within enamel under light exposed conditions.

Enamel	Exposed to Light (Wavelength 410–460 nm)						*p*-Value
	SDF		SDF + GSH		SDF + KI		
	Mean	SD	Mean	SD	Mean	SD	
Immediate	4.68 ^a^	1.44	1.20 ^b^	0.34	4.65 ^a^	0.84	≤0.001 *
3 h	14.17 ^a^	3.05	2.17 ^c^	0.58	5.28 ^b^	0.71	≤0.001 *
6 h	21.95 ^a^	3.53	3.32 ^b^	0.71	5.51 ^b^	0.81	≤0.001 *
24 h	34.52 ^a^	1.93	7.62 ^b^	2.11	6.23 ^b^	0.86	≤0.001 *
48 h	40.32 ^a^	2.40	9.76 ^b^	2.29	6.55 ^c^	1.06	≤0.001 *
72 h	42.62 ^a^	2.38	10.94 ^b^	2.07	6.90 ^c^	1.42	≤0.001 *
7 days	43.81 ^a^	3.27	13.03 ^b^	2.46	7.08 ^c^	1.97	≤0.001 *
10 days	45.68 ^a^	4.06	13.98 ^b^	2.54	6.88 ^c^	1.24	≤0.001 *
14 days	46.77 ^a^	3.45	14.76 ^b^	2.35	6.99 ^c^	1.39	≤0.001 *

Means with the same letter (a, b or c) within each row are not significant (NS), and (*) means significant at *p* > 0.05.

**Table 2:** Mean and SD for ΔE for different tested groups at all time intervals within enamel under dark conditions.

Enamel	Dark						*p*-Value
	SDF		SDF + GSH		SDF + KI		
	Mean	SD	Mean	SD	Mean	SD	
Immediate	2.63 ^b^	0.88	2.01 ^b^	0.52	4.36 ^a^	1.03	≤0.001 *
3 h	2.79 ^b^	0.99	1.64 ^b^	0.54	5.29 ^a^	1.09	≤0.001 *
6 h	2.61 ^b^	0.83	2.61 ^b^	0.89	5.36 ^a^	1.01	≤0.001 *
24 h	4.66 ^ab^	1.66	3.23 ^b^	0.92	5.68 ^a^	1.28	0.009 *
48 h	5.65 ^ab^	1.91	4.13 ^b^	0.67	6.13 ^a^	1.23	0.033 *
72 h	6.95	2.37	4.95	1.02	6.46	1.50	0.117 NS
7 days	8.35	2.67	5.86	1.53	6.25	1.58	0.134 NS
10 days	11.02 ^a^	2.39	6.55 ^b^	1.20	6.74 ^b^	1.60	0.003 *
14 days	11.87 ^a^	2.32	7.45 ^b^	1.56	6.91 ^b^	1.73	0.004 *

Means with the same letter (a, b or ab) within each row are not significant (NS), and (*) means significant at *p* > 0.05.

**Table 3:** Mean and SD for ΔE for different tested groups at all the time intervals within dentin under light exposed conditions.

Dentin	Light						*p*-Value
	SDF		SDF + GSH		SDF + KI		
	Mean	SD	Mean	SD	Mean	SD	
Immediate	2.47 ^b^	0.57	3.38 ^a^	1.18	4.50 ^a^	1.00	0.002 *
3 h	8.43	1.23	7.12	0.74	7.82	1.42	0.095 NS
6 h	9.65 ^a^	1.32	8.17 ^b^	1.09	8.33 ^b^	1.00	0.016 *
24 h	14.40 ^a^	1.67	11.50 ^b^	1.12	9.12 ^c^	0.92	≤0.001 *
48 h	19.16 ^a^	1.72	12.98 ^b^	1.10	10.42 ^c^	0.73	≤0.001 *
72 h	21.30 ^a^	1.21	15.36 ^b^	0.71	11.60 ^c^	0.99	≤0.001 *
7 days	23.91^a^	1.94	18.55 ^b^	0.77	13.04 ^c^	0.83	≤0.001 *
10 days	27.94 ^a^	1.18	21.63 ^b^	0.58	12.74 ^c^	0.72	≤0.001 *
14 days	27.52 ^a^	1.15	25.67 ^b^	0.45	13.96 ^c^	1.12	≤0.001 *

Means with the same letter (a, b or c) within each row are not significant (NS), and (*) means significant at *p* > 0.05.

**Table 4:** Mean and SD for ΔE for different tested groups at all of the time intervals within dentin under dark conditions.

Dentin	Dark						*p*-Value
	SDF		SDF + GSH		SDF + KI		
	Mean	SD	Mean	SD	Mean	SD	
Immediate	2.05	0.61	2.99	0.91	2.69	1.43	0.129 NS
3 h	6.12	2.18	5.14	1.15	6.57	1.64	0.268 NS
6 h	8.45 ^a^	0.84	5.52 ^b^	1.28	8.00 ^a^	1.04	≤0.001 *
24 h	10.30 ^a^	0.80	7.72 ^c^	0.73	8.84 ^b^	0.86	≤0.001 *
48 h	11.64 ^a^	1.04	9.87 ^b^	0.84	9.64 ^b^	0.55	≤0.001 *
72 h	13.32 ^a^	1.11	12.36 ^b^	0.66	10.69 ^c^	0.76	≤0.001 *
7 days	15.96 ^a^	0.57	15.69 ^a^	0.91	12.54 ^b^	0.91	≤0.001 *
10 days	19.32 ^a^	0.54	18.62 ^b^	0.37	12.84 ^c^	0.85	≤0.001 *
14 days	22.29 ^a^	00.81	20.90 ^b^	0.82	13.61 ^c^	1.02	≤0.001 *

Means with the same letter (a, b or c) within each row are not significant (NS), and (*) means significant at *p* > 0.05.

**Table 5:** Materials.

Code	Materials	Composition	Manufacturer
SDF	Saforide	38% Silver diamine fluoride	Bee Brand Medico Dental, Osaka, Japan
		F = 44,880 ppm, Ag = 253,870 ppm	
KI	Potassium Iodide (KI)	White, crystalline powder of KI	Wako Pure Chemical Industries, Osaka, Japan
GSH	Glutathione	l-Glutathione reduced	Sigma-Aldrich, St. Louis, MO, USA
